# Anti-angiogenesis and anti-metastasis effects of Polyphyllin VII on Hepatocellular carcinoma cells in vitro and in vivo

**DOI:** 10.1186/s13020-021-00447-w

**Published:** 2021-05-31

**Authors:** Chao Zhang, Qingrui Li, Guozheng Qin, Yi Zhang, Chaoying Li, Liwen Han, Rongchun Wang, Shudan Wang, Haixia Chen, Kechun Liu, Chengwei He

**Affiliations:** 1 Beijing University of Chinese Medicine, Beijing, 100102 China; 2 State Key Laboratory of Quality Research in Chinese Medicine, Institute of Chinese Medical Sciences, University of Macau, Macao, 999078 China; 3 Biology Institute, Qilu University of Technology (Shandong Academy of Sciences), Jinan, 250103 China; 4 Aerospace Central Hospital, Beijing, 100049 China; 5 Yunnan Provincial Hospital of Traditional Chinese Medicine/The First Affiliated Hospital of Yunnan University of Chinese Medicine, Kunming, 650021 China; 6 School of Pharmaceutical Sciences, Changchun University of Chinese Medicine, Changchun, 130117 China; 7 Institute of Materia Medica, Shandong First Medical University & Shandong Academy of Medical Sciences, Jinan, 250062 China; 8 Tianjin Key Laboratory for Modern Drug Delivery & High-Efficiency, School of Pharmaceutical Science and Technology, Tianjin University, Tianjin, 300072 China

**Keywords:** Polyphyllin VII, Hepatocellular carcinoma cells, Anti-angiogenesis, Anti-metastasis, NF-κB/MMP-9/VEGF pathway

## Abstract

**Background:**

Polyphyllin VII (PP7), a steroidal saponin from *P. polyphylla* has been found to exert strong anticancer activity. Little is known about the anti-angiogenesis and anti-metastasis properties of PP7. In this study, the anti-angiogenic and anti-metastatic effects of PP7 on HCC and the molecular mechanisms were evaluated.

**Methods:**

Effect of PP7 on angiogenesis was assessed by tube formation assay and applied a transgenic *Tg(fli1:EGFP)* zebrafish model. Effects of PP7 on tumor metastasis and invasion were examined in cell migration and invasion assay, zebrafish tumor xenograft models and lung metastasis mouse models. The protein levels were examined by Western blotting.

**Results:**

PP7 significantly decreased the tube formation of human umbilical vein endothelial cells, the number and length of ISVs and SIVs of transgenic zebrafish, and the metastasis and invasion of cancer cells in vitro and in vivo. The anti-angiogenic and anti-metastatic effects of PP7 in HepG2 cells were attributable, at least partially, to downregulated NF-κB/MMP-9/VEGF signaling pathway.

**Conclusion:**

This study demonstrates that PP7 possesses strong anti-angiogenesis and anti-metastasis activities, suggesting that PP7 could be a potential candidate agent for HCC treatment.

## Background

Hepatocellular carcinoma (HCC), also called malignant hepatoma, the most common primary malignant tumor of the liver, is one of the leading cause of cancer-related mortality and fifth common cancer worldwide [1]. Despite impressive advancement in the treatment of HCC, such as chemotherapy, surgery (liver transplantation or resection), embolization and ablation [2], the long-term prognosis of HCC still remains poor because of high incidences of metastasis and recurrence [3]. Liver metastasis, accounting for one-third of all tumor metastasis, is difficult to treat in the clinic and generally the major cause of death for liver cancer patients [4]. Hence, anti-metastatic therapy has been widely investigated as a promising approach for HCC therapy. Furthermore, tumor progression is highly dependent on angiogenesis, which promotes tumor growth by supplying nutrients and oxygen, meanwhile, facilitating tumor invasion and metastasis [5]. Therefore, angiogenesis is another promising target for antitumor drug development.

The rhizome of *Paris polyphylla* var. *yunnanensis*, or called *Rhizoma Paridis*, a long been used herb in traditional Chinese medicine (TCM), possesses multiple pharmacological activities [6]. Notably, it has been used to treat liver cancer in China for many decades [7, 8]. Phytochemical study showed that *Rhizoma Paridis* mainly contains *Rhizoma Paridis* saponins (RPS), which are the main active ingredient for anticancer treatments [9–12]. Polyphyllin VII (PP7), a pennogenyl saponin and an active component of RPS, has been found to show potent anticancer activities via suppression of proliferation, cell cycle arrest, and induction of apoptosis and autophagy in a wide variety of cancer cell lines in vitro [13–19], and strong anti-inflammatory properties in multiple in vitro and in vivo models [20]. Wang et al. found that PP7 can inhibit endothelial cell migration, invasion, and angiogenesis in vitro [21]. However, the anti-angiogenic and anti-metastatic effects of PP7 on HCC and the underlying mechanisms are poorly explored and will be elucidated in the present study using both in vitro and in vivo models.

The zebrafish (*Danio rerio*) is a freshwater tropical fish and member of the minnow family, and has been an excellent experimental model for ontogenetic development and studies of human diseases and toxicology. Notably, zebrafish has been increasingly recognized as a promising animal model for cancer research [22]. Previous studies have demonstrated that the zebrafish tumor xenograft model could provide reliable and rapid testing of various anti-cancer candidates that inhibit cancer cell proliferation, invasion and metastasis and has the potential to contribute to the discovery of novel anticancer agents [23, 24]. Direct injection into the yolk sac itself is technically easy and is an approach of introduction of cancer cells into zebrafish. The zebrafish develop tumors with similar gene-profiling and histopathological features as human tumors [25]. The zebrafish xenograft model has been used previously to permit xenotransplantation with human cancer cells, including liver, breast, prostate and leukemia cancer cells, without immunosuppression [26, 27], because the adaptive immune system in embryonic zebrafish does not reach maturity until four weeks post fertilization [28]. Moreover, the optical transparency of zebrafish, in combination with various fluorescent cell probes, offer high resolution, noninvasive and rapid live imaging of fluorescently labeled cancer cells in a large number of embryos [29–31]. Furthermore, zebrafish embryos were used for a live in vivo model of angiogenesis because many anti-angiogenic drugs elicit similar responses in this model to those observed in mammalian systems [32] and transgenic zebrafish expressing enhanced green fluorescent protein (EGFP) in the vasculature allow for observation of the responses of live embryos to drugs in real time [33, 34]. In this study, we found, for the first time, that PP7 possessed potent anti-angiogenic and anti-metastatic activities against HCC in transgenic *Tg (fli1:EGFP)* zebrafish model, zebrafish tumor xenograft model and lung metastasis mouse model, which were attributable, at least partially, to the downregulation of the NF-κB/MMP-9/VEGF pathway.

## Materials and methods

### Chemicals and reagents

PP7 was kindly provided by Prof. Zhinan Mei [13, 14] (South-Central University for Nationalities). Roswell Park Memorial Institute (RPMI) 1640, Dulbecco’s modified Eagle’s medium (DMEM), phosphate buffered saline (PBS), and penicillin–streptomycin (PS) were purchased from Gibco (Carlsbad, CA, USA). Fetal bovine serum (FBS) and red fluorescent cell staining chloromethylbenzamido-DiI (CM-DiI) were obtained from Invitrogen (Carlsbad, CA, USA). Dimethyl sulfoxide (DMSO), 3-(4, 5-dimethyl-2-thiazolyl)-2, 5-diphenyl-2H-tetrazolium bromide (MTT), 1-phenyl-2-thiourea (PTU), and tricaine methanesulfonate (MS-222) were purchased from Sigma-Aldrich Co (St. Louis, MO, USA). Cyclophosphamide (CTX) for injection was purchased from Jiangsu Shengdi Pharmaceutical Group Corp (Jiangsu, China). PTK787 was obtained from Novartis (East Hanover, NJ, USA). Matrigel was purchased from BD Biosciences (Bedford, MA, USA). Primary antibodies against NF-κB p65, VEGF, MMP-9, Lamin B, and GAPDH, and secondary antibodies were obtained from Proteintech (Chicago, IL, USA) or Cell Signaling Technology (Danvers, MA, USA).

### Cell culture and drug treatments

Human HCC HepG2 cells, human lung carcinoma A549 cells, and human cervix carcinoma HeLa cells were obtained from American Type Culture Collection (Manassas, VA, USA). Human umbilical vein endothelial cells (HUVECs) were purchased from the Cell Bank of Shanghai Institute of Biochemistry and Cell Biology, Chinese Academy of Sciences (Shanghai, China). Mouse H22 hepatocarcinoma cells were kindly provided by Prof. Qin Fu (Jilin Cancer Hospital). Cells were maintained in RPMI 1640 or DMEM medium including 10% heat-inactivated FBS and 100 units/mL PS and incubated in an atmosphere of 5% CO_2_ at 37 °C. For all in vitro experiments, PP7 powder was dissolved in DMSO to make a stock solution and then freshly diluted in the basal medium. Moreover, the final DMSO concentration in the working solutions was less than 0.1%.

### Cell viability analysis

Cell viability was measured using MTT colorimetric assay. Briefly, HUVECs and HepG2 cells (1 × 10^4^ cells/well) were treated with different concentrations of PP7 for 24 h in 96-well plates. Then, the treated cells were incubated in 0.50 mg/mL MTT solution at 37 °C. After 4 h, the medium was replaced with DMSO to dissolve the formazan crystals. The absorbance at 570 nm was read using a microplate reader and the relative viability of treated cells was expressed as percentage of control untreated cells.

### Cell migration assay

The cell migration capability was performed using the wound healing assay. HUVECs and HepG2 cells were seeded in 12-well plates at 90% confluence were wounded with pipette tips, washed with PBS to remove floating cells, and further incubated in a medium with DMSO or indicated concentration of PP7. The width of the wound line was monitored and photographed using an inverted microscope at 24 h.

### Cell invasion assay

The cell invasion capability was performed using the transwell invasion assay. HUVECs and HepG2 cells were treated with indicated concentrations of PP7 for 24 h, and then transferred into upper chamber and incubated for 24 h at 37 °C. The cells that penetrated to the bottom side of the membrane were fixed in 4% paraformaldehyde and stained with 0.1% crystal violet, and counted the invasing cells under a light microscope.

### Tube formation assay

Effect of PP7 on in vitro angiogenesis was examined in HUVECs. Matrigel was polymerized in a 96-well plate for 30 min at 37 °C. HUVECs were seeded on the surface of the matrigel and treated with indicated concentrations of PP7 for 24 h. The length of formed tubes was observed under a phase-contrast microscope equipped with a digital camera.

### Western blotting

The method of Western blotting was the same as our previous reports [13, 14]. The collected HepG2 cells were lysed with RIPA lysis buffer. Protein concentration was determined by the BCA protein assay kit. Equal amounts of proteins from each group were separated by SDS-PAGE gel electrophoresis, and transferred onto methanol activated PVDF membranes. After being blocked with skimmed milk, the membranes were incubated with primary antibodies, followed by incubation with the corresponding secondary antibodies. The density of the bands was performed with the Image Lab Software (Bio-Rad, Hercules, CA, USA). GAPDH and Lamin B were used as the internal controls.

### Animals

The wild-type AB strain of zebrafish and transgenic *Tg(fli1:EGFP)* zebrafish were used in this study and the animal experiments were performed according to the ethical guidelines of Biology Institute of Shandong Academy of Sciences (Approval No. BISD-20180928). Zebrafish embryos were maintained and raised according to the protocol described previously [35].

Male SPF ICR mice (18–22 g) were purchased from Changchun Institute of Biological Products (Changchun, China). The animals were maintained under pathogen-free conditions, in a 12-h light/dark day cycle at 23 ± 2 °C, and provided standard laboratory diet and water ad libitum throughout the experimental period. All procedures were carried out in accordance with the Guidelines of the Use and Care of Laboratory Animals and approved by the Institutional Ethics Committee of Changchun University of Chinese Medicine (Approval No. CUCM-20170015).

### Toxicity effect of PP7 in zebrafish

Sample toxicity was determined by means of heart beat rate and survival rate of the zebrafish embryos. Briefly, zebrafish embryos (n = 10/group) at 1 h post-fertilization (1 hpf) were transferred into 6-well plates and exposed to different concentrations of PP7. The developmental phenotypes of the experimental embryos were observed every 24 h for 72 h. The survival rates of embryos were measured every day. The heart-beating rate of both atrium and ventricle was measured at 72 h.

### Zebrafish angiogenesis model

Zebrafish transgenic line: *Tg(fli1:EGFP)*, in which the EGFP is expressed in all endothelial cells of the vasculature [36]. Embryos were treated with 0.003% PTU at 6 hpf to prevent pigment formation [37]. Healthy embryos at 24 hpf were transferred randomly into 24-well plates and treated with various concentrations of PP7. All embryos were incubated at 28.5 °C. After 24 h treatment, the embryos were observed for intersegmental vessels (ISVs) development and subintestinal vessels (SIVs) formation by using a microscope with a digital imaging system.

### Xenografts in zebrafish

Zebrafish were used to determine the effects of PP7 on the metastasis of xenografts of cancer cells. Zebrafish xenografts were generated by microinjection of cancer cells labeled with red fluorescent CM-DiI into the yolk sac of zebrafish embryos at 2 day post-fertilization (2 dpf), and zebrafish treated with different concentrations of PP7. Three days after drug administration, live embryos were anesthetized with MS-222 and imaged using a laser scanning confocal microscope. The tumor volume, number of disseminated foci from tumor mass, and maximal distance of metastasis of zebrafish xenografts of HeLa cells, A549 cells and HepG2 cells were obtained, which are representative of the metastasis ability of HeLa cells, A549 cells and HepG2 cells in vivo.

### In vivo* anti-tumor activity in lung metastasis bearing mice*

To establish an in vivo lung metastasis model, H22 cells from mice ascites were suspended at a concentration of 2 × 10^6^ cells/mL with PBS and 0.2 mL was injected through the tail vein of each mice. After injection for 24 h, the mice were randomly divided into 6 groups and were injected intraperitoneally one time daily for 14 days in a volume of 0.2 mL with one of the following treatments: model (normal saline) group, normal (normal saline) group, CTX (20 mg/kg) group, PP7 low dose (0.5 mg/kg) group, PP7 middle dose (1 mg/kg) group, and PP7 high dose (2 mg/kg) group. After the last administration, the mice were sacrificed by cervical dislocation. The lungs with tumor nodes were collected, weighed and placed in Bouin stationary solution (saturated picric acid: formalin: acetic acid = 15:5:1) for 24 h. One day later, the metastasized nodes on the surface of lungs were imaged by a digital camera and counted to evaluate the therapeutic effects of PP7.

### Statistical analysis

Data were expressed as the mean ± standard error of the mean (SEM) or standard deviation (SD). The comparison of survival curves was analyzed using the log-rank test. One-way analysis of variance analysis with Tukey post hoc was used to compare differences in variables of different treatment groups. Statistical analysis was performed using GraphPad Prism software (La Jolla, CA, USA). The value of statistical significance is set at *P* < 0.05.

## Results

### Effects of PP7 on viability in HUVECs and HepG2 cells

**Fig. 1:**
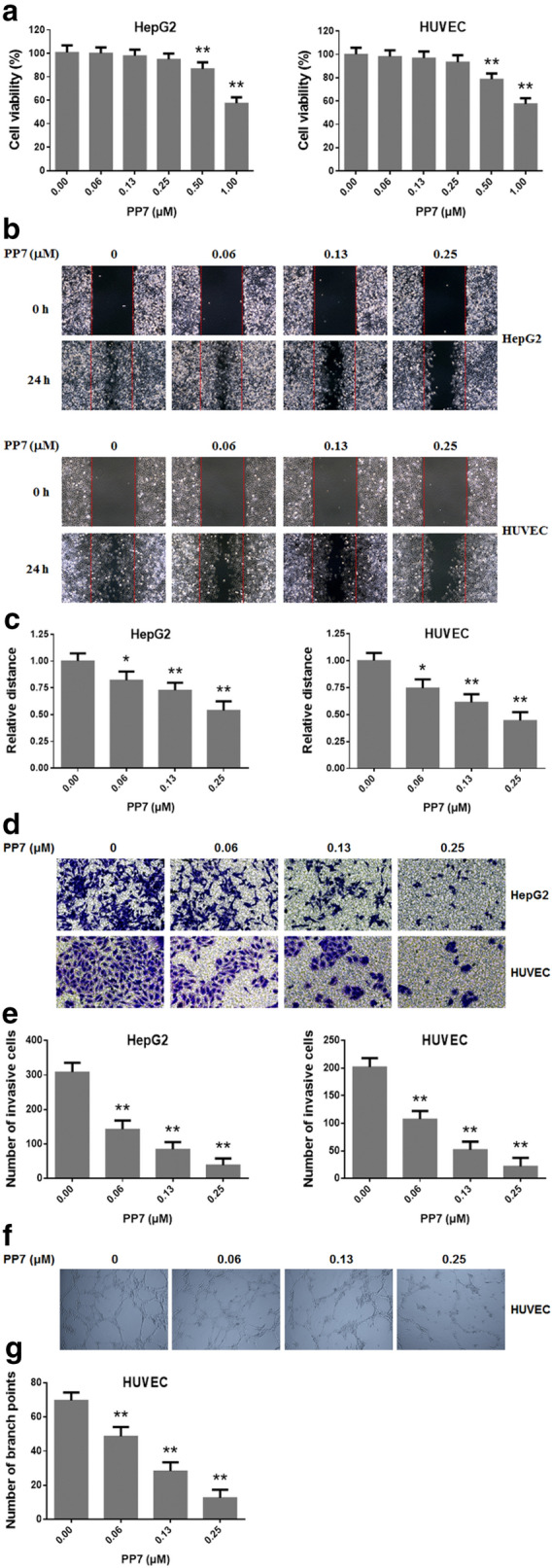
Effects of PP7 on cell viability, motility, invasion, and endothelial tube formation in vitro. **a** HepG2 and HUVECs cells were treated with indicated concentrations of PP7 for 24 h and the cell viability was assessed using an MTT assay. **b** HepG2 and HUVECs cells were scratched with a pipette tip, and the cells were exposed to different concentrations of PP7 for 24 h. **c** Quantification of relative distance of the denuded zone of **b**. **d** HepG2 and HUVECs cells were incubated with various concentrations of PP7 for 24 h. Representative images are shown. **e** Quantification of migrating and invasive cells of **d**. **f** HUVEC cells were plated on Matrigel and treated with increasing concentrations of PP7 for 24 h. Capillary tube formation was observed and photographed at × 200. **g** Quantification of branch points of **f**. Values are expressed as means ± SD of triplicate experiments. *P < 0.05, **P < 0.01, versus control groups

To observe the effects of PP7 on cell viability in HUVECs and HepG2 cells, the cells were treated with different concentrations of PP7 (0.06–1.00 μM) for 24 h and then assessed by MTT assay. PP7 had no effect on cell viability up to a concentration of 0.25 μM (Fig. 1a). Therefore, the non-cytotoxic concentrations of PP7 from 0.06 to 0.25 μM were used in subsequent in vitro experiments.

### Effects of PP7 on the migration and invasion of HUVECs and HepG2 cells

We investigated the inhibitory effects of PP7 on the cell migration and invasion of HUVECs and HepG2 cells using wound-healing and transwell assays. The results indicated that, after 24 h incubation with indicated concentrations of PP7, the migration and invasion of human HCC cells and human endothelial cells were significantly decreased in a dose-dependent manner as compared with control cells (Fig. 1b–e).

### PP7 suppressed tube formation of HUVECs

We examined the effect of PP7 on the angiogenesis of HUVECs cells using a dimensional Matrigel capillary tube formation assay to mimic in vivo angiogenesis. The results indicated that PP7 block angiogenesis in vitro by inhibiting the formation of tubular structure, consisting of the elongation and alignment of the endothelial cells at the indicated concentrations of PP7 (Fig. 1f, g).

### Effect of PP7 on the NF-κB/MMP-9/VEGF pathway in HepG2 cells

**Fig. 2:**
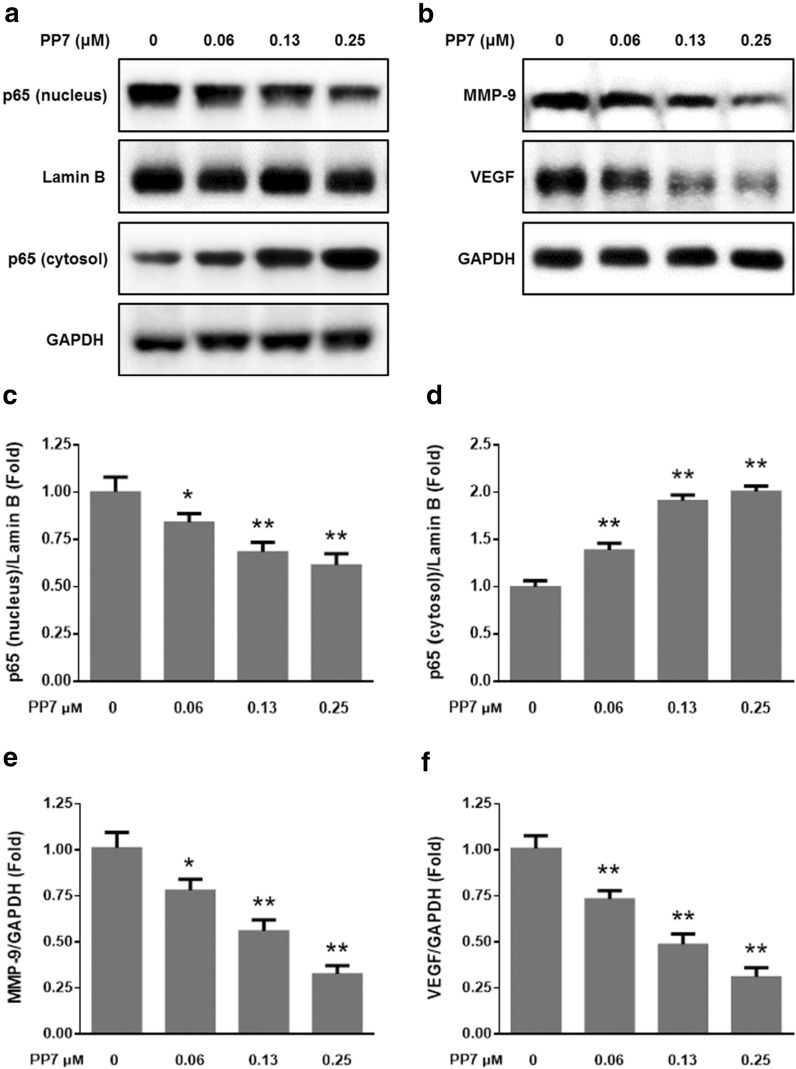
Effect of PP7 on the NF-κB/MMP-9/VEGF pathway in HepG2 cells. **a** Western blot analysis for NF-κB p65 in the nuclear and cytoplasmic extracts of HepG2 cells. (B) MMP-9 and VEGF protein levels were determined via Western blotting. **c**, **d** were densitometric analysis of **a**. **e**, **f** were densitometric analysis of **b**. Values are expressed as means ± SD of triplicate experiments. *P < 0.05, **P < 0.01, versus control groups

Since NF-κB/MMP-9/VEGF pathway play pivotal roles in tumor angiogenesis and metastasis [38, 39]. We examined the protein levels of nuclear factor kappa B (NF-κB), matrix metalloproteinase-9 (MMP-9), and vascular endothelial growth factor (VEGF) in HepG2 cells treated with PP7 by Western blotting assay. As shown in Fig. 2, the results showed that the levels of nuclear NF-κB p65 decreased while cytosolic NF-κB p65 increased markedly after treatment with PP7. Moreover, PP7 evidently decreased MMP-9 and VEGF expression at the protein level in HepG2 cells. These results indicated that NF-κB/MMP-9/VEGF pathway was involved, at least partially, in the anti-angiogenesis and anti-metastasis effects of PP7 on HepG2 cells.

### Effects of PP7 on morphological changes, heart beat rate and survival rate in zebrafish embryo

**Fig. 3:**
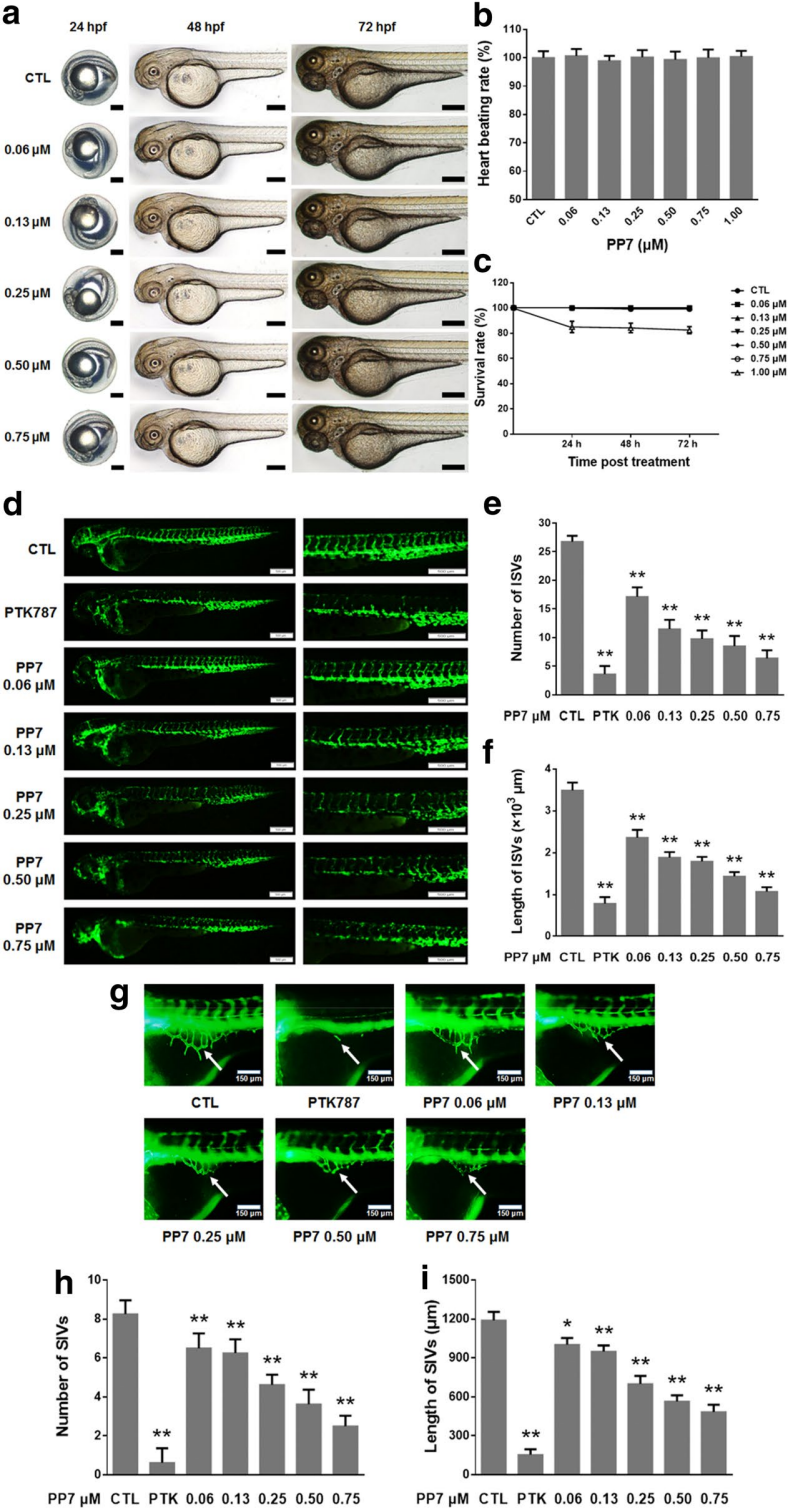
Effects of PP7 on morphological changes, heart beat rate, survival rate, ISVs development and SIVs formation in zebrafish embryos. **a** The morphological features of zebrafish embryos observed and captured after treating embryos with PP7 for 24, 48 and 72 h. Scale bars represent 200 μM. **b** The average heart beating rates per minute of PP7 treated embryos were recorded at 72 h. **c** Survival rates of zebrafish embryos after treatment with PP7 for 24, 48 and 72 h. Lateral view of transgenic *Tg(fli1:EGFP)* zebrafish showing ISV (**d**) and SIV (**g**) growth after treatment for 24 h. Scale bars represent 500 μm (**d**) or 150 μm (**g**). Arrow showed the characteristic pattern of SIV. **e** and **f** was statistical analysis of number and length of ISVs of **d**, respectively. **h**, **i** was statistical analysis of number and length of SIVs of **g**, respectively. *n* = 10/group, *P < 0.05, **P < 0.01 as compared with control

In order to determine the toxicity of PP7 in zebrafish model, we observed the morphological changes, heart beat rate and survival rate in zebrafish embryos. As shown in Fig. 3a, there is no morphological change in zebrafish embryos compared to the control indicating that there is no toxicity at the tested concentrations. Moreover, there is no significant change in heart beat rate (Fig. 3b). In addition, almost the same trend was observed in the survival rates at the sample concentrations tested. However, a slight decrease in the survival rate was observed at the concentration of 1 μM (Fig. 3c). For this reason, 0.75 μM was selected as the highest safe concentration for further experiments in zebrafish.

### Effects of PP7 on angiogenesis in zebrafish embryos

To evaluate the effect of PP7 on angiogenesis, we employed the transgenic *Tg(fli1:EGFP)* zebrafish model, which expresses the EGFP in blood vessels. The results showed that PP7 treatment significantly decreased the number and length of ISVs of zebrafish embryos in a dose-dependent manner (Fig. 3d–f), which was similar to the activity of positive control agent PTK787, which is an angiogenesis inhibitor targeting VEGF receptor tyrosine kinases.

Subsequently, we found that PP7 had an evident effect on SIV formation. The number and length of SIV were remarkably reduced following treatment with PP7 at concentrations from 0.06 μM to 0.75 μM, compared to the control group in a dose–dependent manner (Fig. 3g–i). These results demonstrate that PP7 is an effective angiogenesis inhibitor.

### Effects of PP7 on the metastasis of cancer cells in zebrafish tumor xenografts

**Fig. 4:**
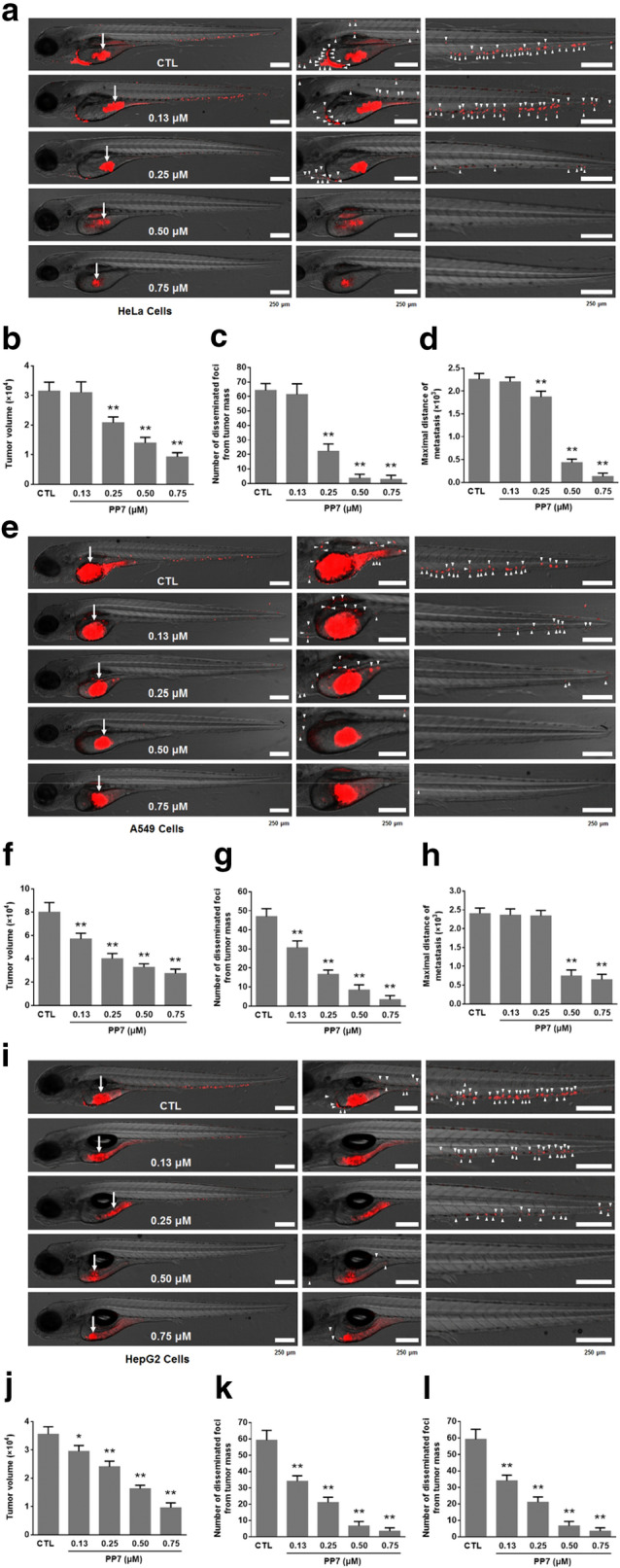
Inhibitory effects of PP7 on the invasion, dissemination and metastasis of HeLa cells, A549 cells and HepG2 cells in zebrafish xenografts. CM-Dil-labeled (red) HeLa cells (**a**), A549 cells (**e**) and HepG2 cells (**i**) were microinjected into the yolk sac of 2dpf embryos, and treated with different concentrations of PP7. Tumor cell invasion, dissemination and metastasis were detected using a laser scanning confocal microscope at day 3 post-injection. Arrows indicate primary tumors, white arrowheads indicate disseminated tumor foci. Scale bars represent 250 μm. Quantification of tumor volume of HeLa cells (**b**), A549 cells (**f**) and HepG2 cells (**j**). Quantification of numbers of disseminated tumor foci of HeLa cells (**c**), A549 cells (**g**) and HepG2 cells (**k**). Averages of maximal distances of metastatic foci of HeLa cells (**d**), A549 cells (**h**) and HepG2 cells (**l**). Data are represented as means ± SD from 3 independent experiments. *n* = 10/group, *P < 0.05, **P < 0.01 as compared with control

We determined the effect of PP7 on the metastasis of HeLa cells, A549 cells and HepG2 cells in zebrafish xenografts. After 72 h xenotransplantation, a massive number of HeLa cells, A549 cells and HepG2 cells migrated to distant parts of the zebrafish body in the control group, while cancer cells in the PP7-treated groups did not migrate far from the primary site. Our results showed that the xenografts of HeLa cells, A549 cells and HepG2 cells treated with PP7 had smaller tumor volume, number of disseminated foci form tumor mass, and maximal distance of metastasis than that in the control group, indicating that PP7 exhibits inhibitory effects on the invasion, dissemination and metastasis of HeLa cells (Fig. 4a–d), A549 cells (Fig. 4e–h) and HepG2 cells (Fig. 4i–l) in zebrafish xenografts.

### Effects of PP7 on metastasis in H22 lung metastasis mouse model

**Fig. 5:**
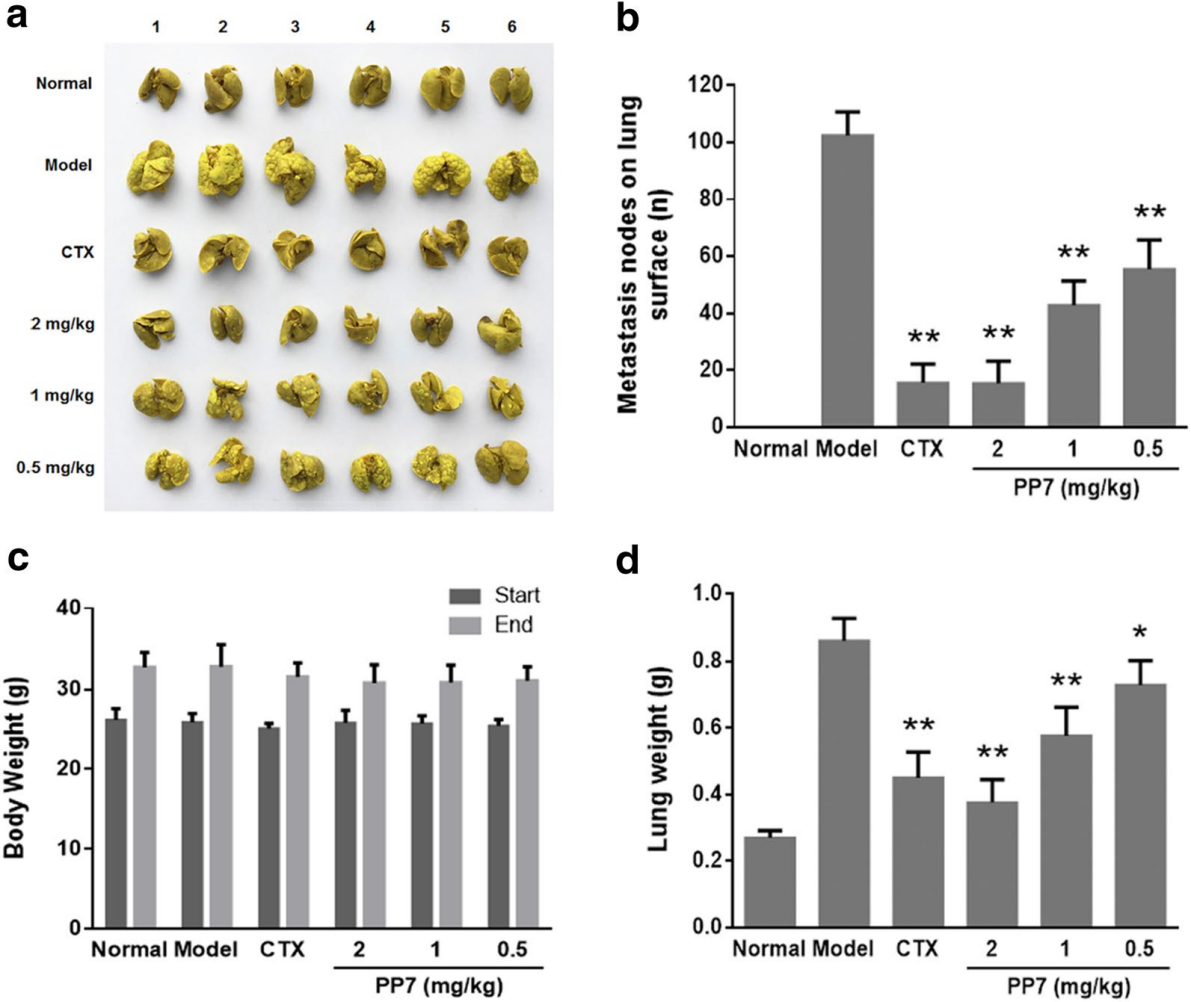
Effects of PP7 on metastasis in H22 lung metastasis mice model. After 14-day treatment, the mice were all euthanized and the lungs were dissected out. **a** The numbers of the lung metastasis nodes were photographed using a camera after fixing with Bouin’s solution. **b** The number of metastasis nodules on the lung surface. **c** The body weights of tumor-bearing mice before and after the 14-day treatment. **d** Lung weights of the mice at the end of the study. Values represent the mean ± SD, *n* = 6. *P < 0.05 and **P < 0.01 were compared with model group

An H22 pulmonary metastasis mouse model was applied to evaluate the ability of PP7 in the inhibition of cancer cell metastasis in vivo. The results are shown in Fig. 5a, b, a large number of H22 metastasis colonies have occupied the lung tissues of the mice in the model group, with an average number of metastasis nodes of 95.83. In contrast, this number was respectively decreased in PP7 groups (0.5, 1, and 2 mg/kg) to 57.17, 42.83 and 15.50 and CTX group to 16.17 without obvious changes in the body weights of the mice (Fig. 5c).

As shown in Fig. 5d, the average weight of lung tissues in different groups reveals that the model group have a much heavier lung tissue (about 0.67 g) than that in healthy mice (about 0.28 g), resulting from the malignant edema of lung tissues and growth of H22 metastasis colonies. However, the lung tissue of mice in PP7 (2 mg/kg) and CTX treated groups shows an approximate weight of about 0.35 g and 0.33 g, respectively. These results indicated that PP7 significantly inhibited H22 cancer cell metastasis and improved the lung injury caused by the metastasis.

## Discussion

Polyphyllin VII (PP7), an active steroidal saponin purified from *Rhizoma Paridis*, has been identified to exert potent anticancer effects in vitro via suppression of cell proliferation, cell cycle arrest, and induction of apoptosis and autophagy [13–19], and possess strong anti-inflammatory activities in vitro and in vivo [20]. In this study, we firstly demonstrated that PP7 exhibited anti-tumor activities via inhibiting angiogenesis and metastasis in vitro and in vivo.

Metastasis is a hallmark of malignant diseases and causes the death of the vast majority of cancer patients [40]. Liver metastasis is difficult to treat in the clinic and generally causes the death of cancer patients [4]. Metastasis is a multistage process involving dissemination of cancer cells from primary sites, transport of cancer cells via the circulation or lymphatic system to distal organs and tissues, formation of metastatic niches, and re-growth of cancer cells into a detectable mass [41, 42]. Furthermore, tumor metastasis can occur at the early stage of malignant diseases when primary tumors are relatively small and a substantial number of patients are diagnosed for malignant diseases due to metastasis as the first sign of cancer [43]. Once a solid tumor spreads to other organs and tissues, surgical operation, intervention with cancer drugs, and radiotherapy are usually ineffective [44]. Therefore, anti-metastasis has been widely investigated as a promising approach for cancer therapy and is crucial for improvement of life quality and survival time of cancer patients. In this study, we observed that the motility and invasion ability of HCC cells was reduced by PP7 treatment in vitro (Fig. 1b–e). Moreover, we determined the anti-metastatic activity of PP7 using a zebrafish metastatic model that allows us to monitor dissemination of cancer cells from primary sites by visualizing single cell invasion and metastasis in vivo without invasive procedures. Zebrafish metastatic model is complementary to existing mice metastatic models, allowing the study of different aspects of tumor metastasis. In our results, PP7 significantly inhibited the invasion, dissemination and metastasis of human cancer cells in zebrafish xenografts (Fig. 4). Anti-metastasis effect of PP7 was also studied using H22 lung metastasis mouse model, which could develop spontaneous pulmonary metastasis [45]. In the present study, we found that PP7 inhibited lung metastases of H22 and improved the lung injury caused by the metastasis of H22 in mice in a dose-dependent manner (Fig. 5). These results demonstrated that PP7 exhibits potent anti-metastatic activities in vitro and in vivo.

Angiogenesis, the formation of new capillaries via branching from the existing vasculature, is closely associated with solid tumor growth and tumor metastatic ability [46]. Angiogenesis is especially essential for the growing tumor because solid tumors require sufficient supply of nutrients from the new blood vessels to grow and metastasize [47], which is particularly fast in HCC [2]. Recently, angiogenesis inhibition has received increased attention as a novel approach for cancer therapy [48]. Numerous agents already in clinical trials have been shown to possess antiangiogenic activity. However, the effect of PP7 on angiogenesis remains unknown. In this study, we showed that PP7 reduced tube formation of endothelial cells in vitro (Fig. 1f, g). Furthermore, we applied transgenic zebrafish *Tg(fli1:EGFP)*, which allows direct visualization of vascular development without any mechanical disruption, to investigate the effect of PP7 on angiogenesis. In comparison with control group, the number and length of ISVs and SIVs in PP7-treated groups were significantly inhibited (Fig. 3). These results suggest that PP7 inhibits tumor growth and metastasis by inhibiting angiogenesis.

The NF-κB/MMP-9/VEGF pathway plays pivotal roles in the tumor progression, angiogenesis, and metastasis. As a transcription factor with multidirectional regulatory function, NF-κB plays an important role in cell carcinogenesis and regulates the expression of its downstream target genes including MMP-9 and VEGF, which are closely associated with tumor genesis, metastasis and angiogenesis [38, 39]. NF-κB bound with IκB-α in the cytoplasm as an inactive form under normal conditions. When cells are stimulated by external factors, NF-κB is released from binding with IκB-α, and then enters the nucleus. NF-κB p65 is the major subunit of NF-κB [49]. In the present study, we found that PP7 significantly decreased NF-κB p65 level in the nucleus and enhanced NF-κB p65 level in the cytoplasm in HepG2 cells (Fig. 2a). MMPs are a subfamily of zinc-dependent proteases capable of degrading extracellular matrix components and play vital roles in the invasion and metastasis of malignant tumors. MMP-9 is one of the MMPs and high MMP-9 expression in multiple tumors can stimulate tumor cells to move out of the carcinoma nest and promote tumor cells going in and out of blood vessels, thus enhancing invasion and metastatic abilities of tumors [50]. VEGF, an important multi-functional angiogenesis factor, plays a critical role in angiogenesis, progression and metastasis of tumors. Its high expression in all malignant tumors [51]. In our results, PP7 significantly decreased MMP-9 and VEGF expression in HepG2 cells (Fig. 2b). These data suggest that PP7-inhibited HCC angiogenesis and metastasis were attributable, at least partially, to the downregulation of NF-κB/MMP-9/VEGF pathway.

## Conclusion

In summary, this study demonstrated that PP7 has strong anti-angiogenic and anti-metastatic activities in vitro and in vivo. The anti-angiogenic and anti-metastatic activities of PP7 in HepG2 cells were attributable, at least partially, to downregulated NF-κB/MMP-9/VEGF pathway. This study suggests that PP7 could be a promising therapeutic agent for the management of HCC.

## Data Availability

The datasets used and/or analyzed during the current study are available from the corresponding author on reasonable request.
